# Supplementary material for: Dual Oxidase 2 (DUOX2) as a Proteomic Biomarker for Predicting Treatment Response to Chemoradiation Therapy for Locally Advanced Rectal Cancer: Using High-Throughput Proteomic Analysis and Machine Learning Algorithm
Source: Int J Mol Sci. 2022 Oct 26;23(21):12923. doi: 10.3390/ijms232112923 (PMC9656829; doi:10.3390/ijms232112923)
Supplement: Supplementary file 1 [file ijms-23-12923-s001.zip › ijms-1913954-supplementary.pdf]

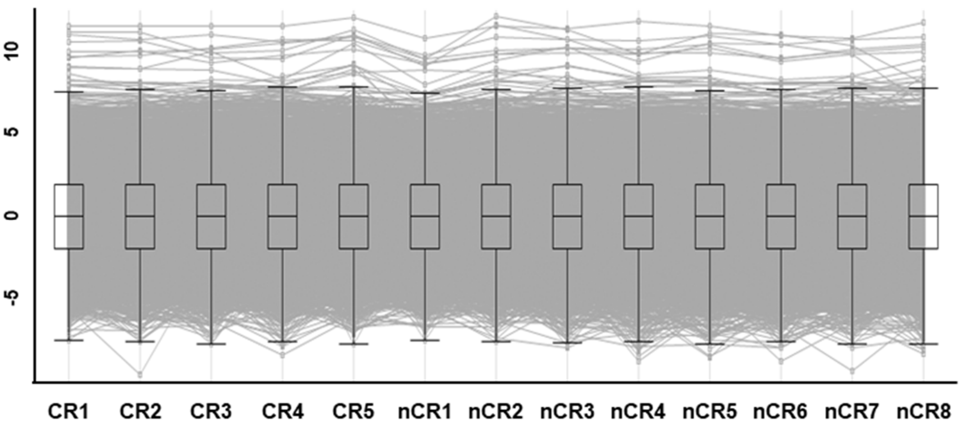

(**A**)

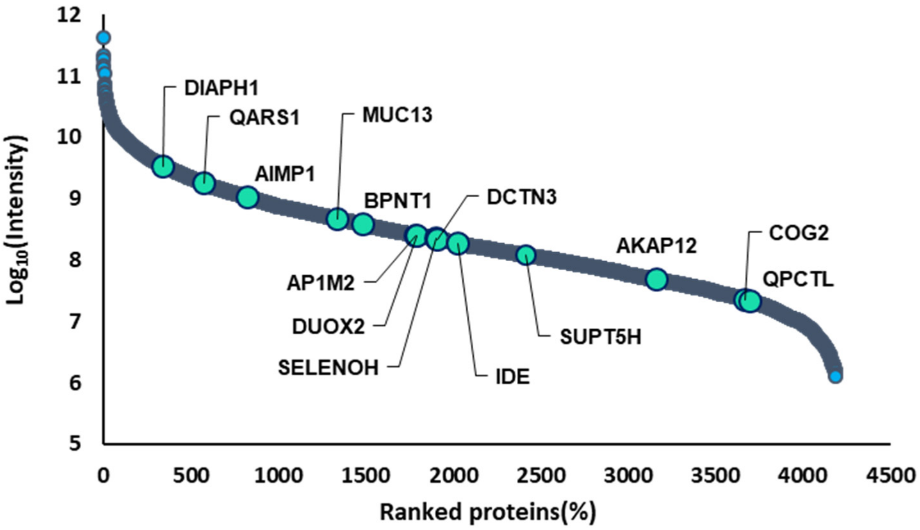

(**B**)

Figure S1. (**A**) **Normalization of protein expression**. Data were normalized using width adjustment for all values in a sample. (**B**) **Dynamic range of quantified proteins.** Distribution of expression intensities of quantified proteins show a large dynamic range of abundance, but most of the proteins were expressed within eight orders of magnitude. Several proteins, including DUOX2, were quantified.
